# The Role of the Insular Cortex in Retaliation

**DOI:** 10.1371/journal.pone.0152000

**Published:** 2016-04-20

**Authors:** Franziska Emmerling, Teresa Schuhmann, Jill Lobbestael, Arnoud Arntz, Suzanne Brugman, Alexander Thomas Sack

**Affiliations:** 1 Department of Cognitive Neuroscience, Maastricht University, Maastricht, The Netherlands; 2 Maastricht Brain Imaging Center, Maastricht, The Netherlands; 3 Department of Clinical Psychological Science, Maastricht University, Maastricht, The Netherlands; 4 Department of Clinical Psychology, University of Amsterdam, Amsterdam, The Netherlands; Chinese Academy of Sciences, CHINA

## Abstract

The insular cortex has consistently been associated with various aspects of emotion regulation and social interaction, including anger processing and overt aggression. Aggression research distinguishes proactive or instrumental aggression from retaliation, i.e. aggression in response to provocation. Here, we investigated the specific role of the insular cortex during retaliation, employing a controlled behavioral aggression paradigm implementing different levels of provocation. Fifteen healthy male volunteers underwent whole brain functional magnetic resonance imaging (fMRI) to identify brain regions involved in interaction with either a provoking or a non-provoking opponent. FMRI group analyses were complemented by examining the parametric modulations of brain activity related to the individual level of displayed aggression. These analyses identified a hemispheric lateralization as well as an anatomical segregation of insular cortex with specifically the left posterior part being involved in retaliation. The left-lateralization of insular activity during retaliation is in accordance with evidence from electro-physiological studies, suggesting left-lateralized fronto-cortical dominance during anger processing and aggressive acts. The posterior localization of insular activity, on the other hand, suggests a spatial segregation within insular cortex with particularly the posterior part being involved in the processing of emotions that trigger intense bodily sensations and immediate action tendencies.

## Introduction

Aggression is defined as behavior intended to inflicting harm to another being, while the victim wants to avoid the harm [1]. Different forms of aggression can be distinguished; proactive aggression refers to using aggression in an instrumental, goal-oriented way, whereas reactive aggression refers to retaliation, i.e. aggressive actions triggered by preceding provocation [2,3].

Aggression and retaliation are complex social behaviors and their scientific assessments rely on social interaction paradigms that do not only measure the perception of—or attention to—specific social emotional cues, but also their behavioral consequences in an experimental setup. Ideally, such paradigms allow quantifying different levels of aggressive behavior within provocative and non-provocative interactions in a controlled way. One of the most widely used and validated behavioral aggression paradigms fulfilling these requirements is the Taylor Aggression Paradigm (TAP [4]), which also proved feasible in an neuroimaging environment [5–11]. The TAP is set up as a reaction time game between two or more opponents in which the winner can administer an aversive feedback stimulus of variable intensity to the opponent. It measures aggressive behavior within direct social interactions in a controlled way. Several neuroimaging studies have aimed at identifying neural activity induced by the TAP, reporting predominantly prefrontal regions, parietal cortex, basal ganglia, thalamus [9], and dorsal medial prefrontal cortex [10] involvement during the interaction between opponents. Higher nucleus accumbens activation was shown to predict more aggressive retaliation [11]. Prefrontal regions, striatum, and other parts of the reward network [9] were activated when winning (versus losing) against the opponent. Furthermore, the insular cortex was especially associated with aggressive behavior [8,9] during this social interaction game. Insular cortex involvement has also been reported in many other contexts including the processing of positive emotions, action inhibition, mindfulness, and interoception [12], questioning any functional specificity of its involvement.

The seemingly rather general involvement of insular cortex in a variety of emotional and cognitive paradigms led to the development of models which could potentially assign different functions to different parts of the insular cortex. For instance, a segregation along a posterior-to-anterior gradient representing the progressive integration of bodily feelings has been proposed [12]: Whereas acute emotions or interoceptive components might be represented in the posterior parts of the insular cortex, the anterior parts seem to code for more abstract and highly integrated constructs. This suggests that—opposite to the anterior insular cortex—the posterior insular cortex is involved in the processing of emotions triggering intense bodily sensation and immediate action tendencies.

Besides its segregation, the lateralization of insular activation is yet to be fully understood. Previous work suggested that the left hemisphere—as opposed to the right—might be involved in approach related motivational states such as anger processing and aggression [13,14]. It remains to be answered, however, whether this assumption holds true not only regarding overall fronto-cortical asymmetry, but also with respect to specific brain regions such as the insular cortex.

Following this line of argumentation, we particularly expected the posterior parts of the insular cortex to be involved in retaliation during which intense bodily sensations, action-oriented emotional content, and immediate behavioral responses are mobilized. Furthermore, this activation was expected to be left-lateralized as retaliation is closely related to anger processing and approach motivation. The here presented study tested this hypothesis by assessing the parametric modulations of brain activity underlying retaliation in a controlled aggression paradigm during whole brain fMRI.

## Materials and Methods

Please note that other analyses based on the same data set were reported previously comparing the neural correlates of reactive aggression with those of motor impulsivity (measured with a go-/nogo task [8]. The neural correlates of the employed aggression paradigm were not yet exhaustively analyzed previously. We, here, therefore focus on the complete analysis of the data related to the aggression paradigm, including the analysis of parametric modulations as well as group analyses of each trial’s entire time course.

### Participants

Eighteen male university students volunteered, gave their written informed consent, and were paid for participating. A screening ensured that none of the participants had a previous history of neurological or psychiatric disorders. Data of two participants were excluded from the analyses as they did not follow the instructions of the experimenter and chose fixed button press pattern without considering the actual task. Data of another participant was excluded, since the participant did not show any variance in behavior and always chose the exact same answer. Data of fifteen participants were included in further analyses (mean age = 22.33; SD = 2.35). The study was approved by the local Ethical Committee of the Faculty of Psychology and Neuroscience at Maastricht University and has been conducted according to the principles expressed in the Declaration of Helsinki.

### Taylor Aggression Paradigm (TAP)

Introduced in its first version by Taylor in 1967 [4], the TAP has become a common tool in behavioral aggression research and has also proven itself the most adaptable option for brain imaging studies [5,6,8–10]. The task is set up as a competitive reaction time game between two or more opponents. The players give each other feedback in form of an aversive noise stimulus after each reaction time trial. During the task, aggressive behavior is measured by recording the severity level of the feedback or retaliatory aggression participants assign to their virtual opponents. The level of provocation can be manipulated such that an opponent can choose a more or less aversive feedback for the other player. Whenever a player loses a reaction time trial, he is presented with the aversive noise chosen by the opponent. The Taylor Aggression Paradigm has shown to be high in construct, internal, discriminant as well as external validity [15–17].

During recruitment, participants were led to believe that the experiment investigated the impact of human feedback on reaction time performance. They were informed about playing a reaction time game (TAP) against two other participants. Before entering the scanner, the participant and the two opponents (collaborators of the experimenters) were introduced. The experimenter’s collaborators were trained beforehand and acted according to a script in order to ensure equal treatment of all participants. Throughout the entire scan, the players communicated verbally via intercom between the experimental runs. Immediately after completion of the experiment, an exit interview was administered to ensure that participants were fully deceived by the experimental setup. Upon completion of the study, a written debriefing was provided.

**Fig 1:**
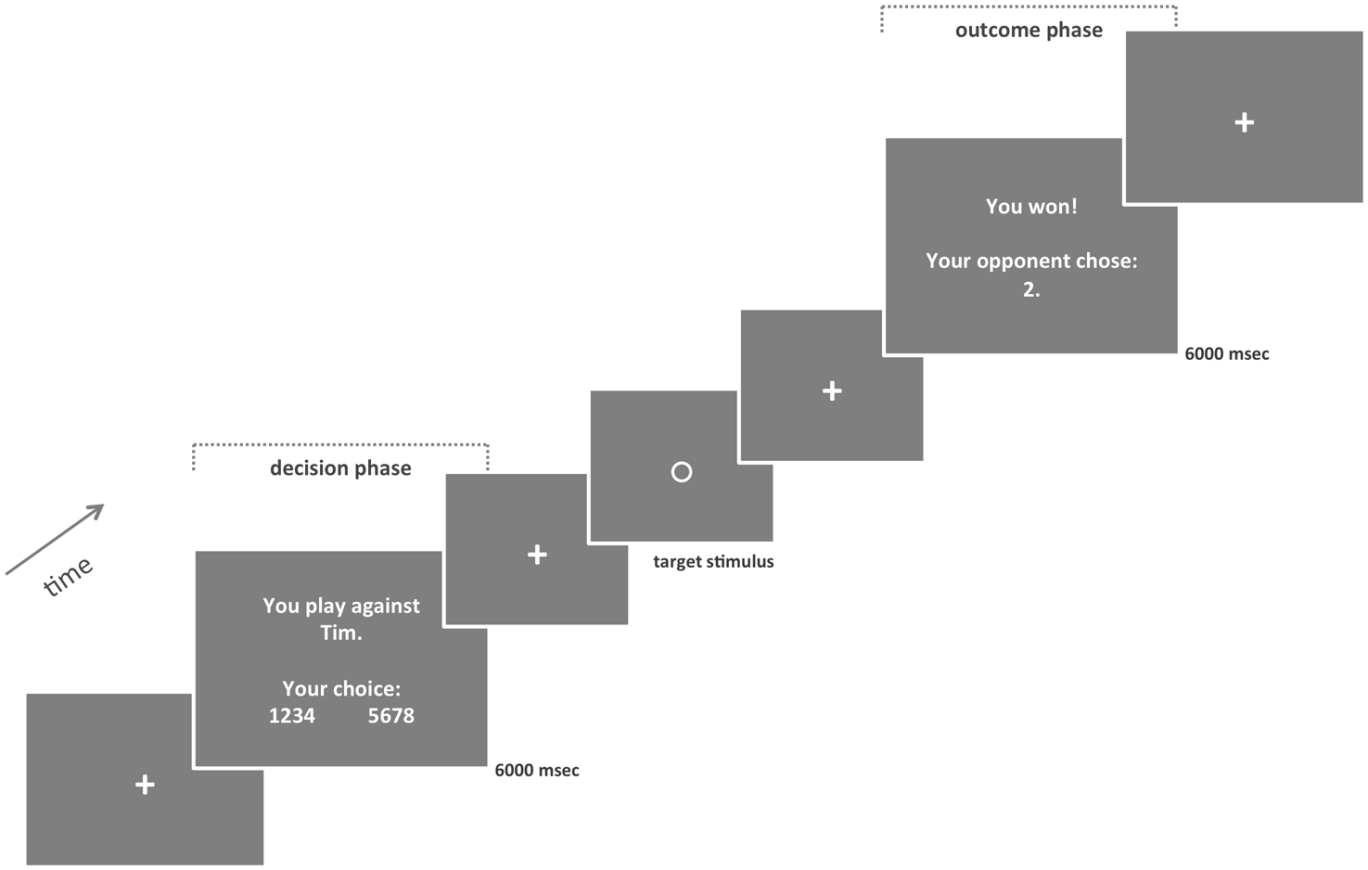
Taylor Aggression Paradigm (TAP). Adapted from Dambacher et al., 2014 [8]. During the decision phase, participants were presented with a screen that informed them against whom they were playing in this trial (in this case “Tim”) and asked to choose the feedback noise level that should be administered to this opponent in case the opponent lost (“12345678”). During the outcome phase, participants were informed on whether or not they won and what feedback noise level the particular opponent had chosen for this trial.

In the implementation of the TAP employed in this study [8], Fig 1), the participant played reaction time trials against one of two alleged opponents. These opponents were collaborators of the experimenter and merely acted in their role as participants. Participants were told that whoever reacted faster to a target stimulus, won the trial. The loser of each trial was presented with an aversive feedback noise chosen by the winning opponent. At the beginning of each trial the volume of this noise was chosen on an 8-point scale. Feedback noises were adjusted to the individual threshold of endurability while running a functional sequence for each participant (a 10 noise was set to the loudness the participant reported subjectively as the ultimate limit of endurability). No noises above 100 decibel were administered to ensure that the hearing was not compromised. Participants randomly played against each of the putative opponents in 50% of the trials. One opponent always selected soft feedback noises (from 1 to 4; non-provoking opponent), while the other selected loud feedback noises (from 4 to 8; provoking opponent). Participants randomly won (and lost) in 50% of trials per opponent. Trials in which reaction times exceeded 500 msec always were losing trials. This ensured a realistic sensation of competing with a human opponent.

Each trial of 27000 msec consisted of a decision phase (6000 msec), the actual reaction time game (jittered between 4500 and 7500 msec), and an outcome phase (6000 msec). A jittered resting period followed. During the decision phase, participants were presented with a screen that informed them against whom they were playing in this particular trial (“Rob” or “Tim”) and asked to choose the volume of the noise feedback they wanted to administer to this opponent in case he would lose. The actual reactive aggressive behavior was measured during the decision phase of the TAP. During the outcome phase, participants were informed about whether or not they lost in this particular trial and which feedback noise levels the particular opponent chose for this trial. Whenever they lost, they were presented with this noise at the end of the outcome phase.

Stimuli were presented in white (RGB 255/255/255; Arial pt 24) on a grey background (RGB 125/125/125). Participants performed 3 runs of the TAP including 28 trials (14 trials per opponent) each, leading to a total of 84 trials (42 trials per opponent). Stimuli were presented using Presentation software (Neurobehavioral Systems, Inc., Albany, USA). Behavioral statistical analyses were performed using SPSS19 (IBM Statistics, USA).

### Technical details, fMRI acquisition and analysis

See also [8]. Stimulus material was presented using an LCD projector (Panasonic, No PT-EZ57OEL) mounted onto a frosted screen, positioned at rear of the scanner bore. Responses were registered with a standard MR compatible button box (Current Designs, 8-button response device, HHSC-2x4-C, Philadelphia, USA).

Images were acquired with a 3 Tesla Siemens Prisma scanner. Structural (high resolution T1-weighted MPRAGE; isotropic voxel resolution 1x1x1 mm^3^; 192 sagittal slices) and functional whole-brain (Gradient-Echo-EPI-sequence; TR = 1500msec; TE = 26msec; FOV = 224mm; flip angle = 73°; matrix = 64x64; distance factor = 20%; 478 volumes per run for the GNGT, 512 volumes per run for the TAP) scans were recorded. Twenty-eight oblique transversal slices of 3.5x3.5x3.5mm voxels were obtained. Slices were tilted 30° relatively to the anterior-posterior commissure plane to avoid signal dropout in frontal areas [18].

FMRI data were analyzed with Brain Voyager QX (Brain Innovation BV, Maastricht, The Netherlands). Preprocessing included 3D-motion-correction (trilinear / sinc interpolation and intra-session alignment to the first functional volume recorded after the individual structural scan), cubic spline slice scan time correction, and the application of a temporal high pass filter (general linear model (GLM) with Fourier basis set of 3 cycles sine/cosine per run plus linear trend removal). Images were co-registered to the individual anatomical data sets and normalized to Talairach stereotaxic space [19]. Volume time courses were spatially smoothed (6mm full width half maximum Gaussian kernel).

The first three trials per opponent were excluded to restrict the analyses to the trials in which participants were familiar with the distinct behavioral pattern of the two opponents (i.e. provoking versus non-provoking).

### Random effects group analyses

A GLM was defined to analyze the behavior displayed during the decision and the outcome phase in the TAP. For these analyses, the retaliatory aggression displayed by the participants was grouped into low (level 1–3), middle (4 and 5), and high (level 6–8) retaliatory aggression.

The following conditions were included as predictors for the decision phase (for phases of TAP see Fig 1): participant chooses high retaliatory aggression for the provoking opponent, participant chooses low retaliatory aggression for the non-provoking opponent. Some participants rarely or never chose a low or middle retaliatory aggression for the provoking opponent. Furthermore, not every participant chose a middle or high retaliatory aggression for the non-provoking opponent. These conditions could therefore not be taken into account on the level of group analyses.

The following conditions were included as predictors for the outcome phase (for phases of TAP see Fig 1): all win trials, all lose trials, win trials against provoking opponent, lose trials against provoking opponent, win trials against non-provoking opponent, lose trials against non-provoking opponent.

To reduce error variance, one noise regressor consisting of the first eigenvariate time series from cerebrospinal fluid regions and motion artefacts were included into the analyses as covariates. Statistical maps were created using a threshold of p < .001 corrected for multiple comparisons by means of cluster threshold level estimation (1000 Monte Carlo simulation iterations [20]).

### Analyses of parametric modulations

For these analyses, the feedback given by the participants was treated as a continuous linear variable (from 1 to 8). For the decision phase, a main and a parametric predictor for interaction with the provoking opponent and the non-provoking opponent were defined. For the outcome phase, a main and a parametric predictor for winning and losing against the provoking opponent and the non-provoking opponent were defined. Parametric predictors were weighted on a single trial bases according to the behavior the participant displayed (the retaliatory aggression displayed) in the respective trial.

In order to examine which brain regions were modulated by the chosen retaliatory aggression, the conjunction of the main and the parametric effect for each specific condition (decision and outcome phase) was inspected.

Statistical maps were created using a threshold of p < .01 corrected for multiple comparisons by means of cluster threshold level estimation (1000 Monte Carlo simulation iterations [20]).

## Results

### Behavioral data

The average feedback (i.e. aversive noise) selected by the participants for the opponents was of medium intensity (MEAN = 3.54; SD = .04). A significantly higher feedback was chosen for the provoking compared to the non-provoking opponent (provoking opponent: MEAN = 4.52, SD = .64; non-provoking opponent: MEAN = 2.56, SD = 1.17; t = 4.59, p = < .001). During the exit interview at the end of the experiment none of the participants reported doubting the proposed purpose of the study and all fifteen participants reported that they perceived one opponent as more provocative than the other. Twelve participants explicitly reported that they adapted their reaction to that perception (behavioral data previously reported in Dambacher et al. 2014).

### Random effects group analyses

**Fig 2:**
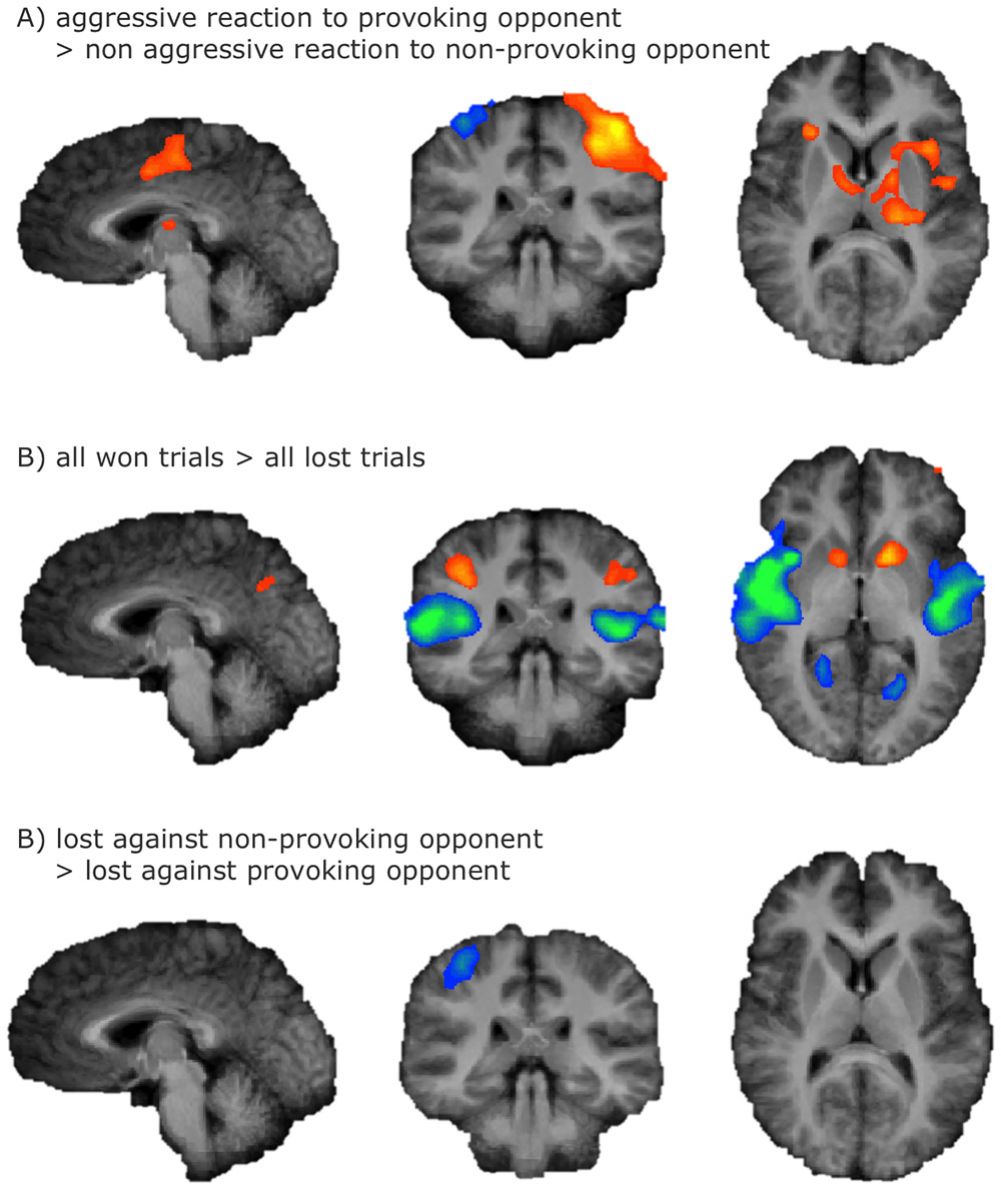
Random effects group analyses. Neural activation for the specified contrasts containing significant activation during the decision phase (A) and the outcome phase (B, C) of the Taylor Aggression Paradigm. Statistical Maps: N = 15, p < .001, Cluster Threshold level corrected, radiological convention.

Talairach coordinates of the brain regions showing increased activation associated with the investigated contrasts are reported in Table 1 (reported are the center of gravity, the number of significant voxels per cluster, and the maximum statistical t-value; cluster are labeled according to Talairach Client [21,22]). Statistical maps of random effects group analyses are depicted in Fig 2 for the decision and the outcome phase.

**Table 1:** Talairach coordinates. Center of gravity, number of significant voxels per cluster, and maximum statistical t-value; clusters are labeled according to Talairach Client [21,22].

		*Talairach coordinates*			*Size*	
*Region*		*x*	*y*	*z*	*voxel*	*t*
**RFX GLM**						
**Aggressive reaction to provoking opponent > non aggressive reaction to non-provoking opponent**						
Anterior insular cortex	R	27	21	10	1126	6.43
Anterior insular cortex *connected*	L	-17	-3	9	13798	7.41
Insular cortex *connected*	L	-17	-3	9	13798	7.41
Putamen / globus pallidus *connected*	R	-17	-3	9	13798	7.41
Putamen / globus pallidus *connected*	L	-17	-3	9	13798	7.41
Thalamus *connected*	L	-17	-3	9	13798	7.41
Caudate *connected*	L	-17	-3	9	13798	7.41
Parietal lobe, postcentral gyrus	L	-38	-34	53	30935	9.96
Frontal Lobe, paracentral lobe	L	-4	-11	47	2900	6.05
Cerebellum	R	12	-65	18	7471	7.87
**Non aggressive reaction to non-provoking opponent > aggressive reaction to provoking opponent**						
Parietal lobe, postcentral gyrus	R	36	-31	60	1796	6.52
Superior temporal gyrus	R	53	4	-7	348	6.04
**Won > lost**						
Superior frontal gyrus	R	21	56	18	1056	5.87
Middle frontal gyrus	L	-44	54	7	449	5.26
Middle frontal gyrus	R	29	8	51	16489	11.67
Middle frontal gyrus	L	-33	6	51	17993	8.20
Inferior parietal lobe, postcentral gyrus	R	38	-56	37	17931	8.07
Inferior parietal lobe, postcentral gyrus	L	-39	-55	37	18740	8.49
Parietal lobe, precuneus	L	0	-63	34	721	5.26
Striatum	R	11	8	3	761	5.77
Striatum	L	-16	10	3	1865	8.07
**Lost > won**						
Superior temporal gyrus	R	48	-15	8	35116	12.37
Superior temporal gyrus	L	-49	-20	8	18628	9.87
Limbic lobe, parahippocampal gyrus	R	18	-50	-2	3383	7.57
Limbic lobe, parahippocampal gyrus	L	-20	-55	-1	1552	6.04
**Won against the provoking opponent > won against the non-provoking opponent**						
*No significant modulation detected*						
**Won against the non-provoking opponent > won against the provoking opponent**						
*No significant modulation detected*						
**Lost against the provoking opponent > lost against the non-provoking opponent**						
*No significant modulation detected*						
**Lost against the non-provoking opponent > lost against the provoking opponent**						
Parietal lobe, around postcentral gyrus	R	35	-32	52	2685	7.64
Parietal lobe, around postcentral gyrus	L	-11	-41	68	1508	
Middle temporal gyrus	L	-41	-64	26	665	6.13
**PARAMETRIC MODULATIONS**						
**Retaliation independent of opponent**						
Inferior parietal lobe, pre- and postcentral gyrus	L	-38	-36	51	29148	6.16
Cerebellum	R	14	-49	-20	1350	3.98
**Retaliation interacting with provoking opponent**						
Inferior parietal lobe, pre- and postcentral gyrus	L	-36	-34	56	17268	6.14
Cerebellum	R	11	-53	-19	1703	4.79
Insular cortex	L	-39	-8	15	1562	6.13
**Retaliation interacting with non-provoking opponent**						
Inferior parietal lobe, pre- and postcentral gyrus	L	-41	-38	47	10864	4.21
**Won**						
*No significant modulation detected*						
**Won against provoking opponent**						
*No significant modulation detected*						
**Won against non-provoking opponent**						
*No significant modulation detected*						

When contrasting trials in which participants displayed high retaliatory aggression towards the provoking opponent with trials in which the participant displayed low retaliatory aggression towards the non-provoking opponent (provocation > no provocation; only contrast previously reported in Dambacher et al. 2014), increased activation in bilateral insular cortex, left parietal lobe, left-lateralized motor regions, the left frontal lobe, and cerebellum was observed. Furthermore, several subcortical regions (i.e., the right and left putamen/globus pallidus, left-lateralized thalamic regions and caudate) showed significant activation change associated to this contrast (Fig 2, Table 1). The only significant activation change associated with the display of low retaliatory aggression towards the non-provoking opponent (no provocation > provocation) was observed in the right parietal lobe close to the postcentral gyrus and the right superior temporal gyrus.

Replicating previous results [9], during winning (all won trials > all lost trials), strong significant bilateral activation in the right superior frontal gyrus, the middle frontal gyri, the left precuneus, the inferior parietal lobes, and the striatum was observed. During losing (all lost trials > all won trials), strong significant bilateral activation in the superior temporal gyri and the parahippocampal gyri was observed. No differences in brain activity could be detected when winning against the provoking opponent as opposed to winning against the non-provoking opponent (won trials against the provoking opponent > won trials against the non-provoking opponent; won trials against the non-provoking opponent > won trials against the provoking opponent). When participants lost to the non-provoking opponent versus to the provoking opponent (lost trials against the non-provoking opponent > lost trials against the provoking opponent), significant bilateral activation in the parietal lobes around the postcentral gyri and the left middle temporal gyrus could be detected. When participants lost to the provoking opponent versus to the non-provoking opponent (lost trials against the provoking opponent > lost trials against the non-provoking opponent), no significant differential brain activity could be detected.

### Parametric modulations

**Fig 3:**
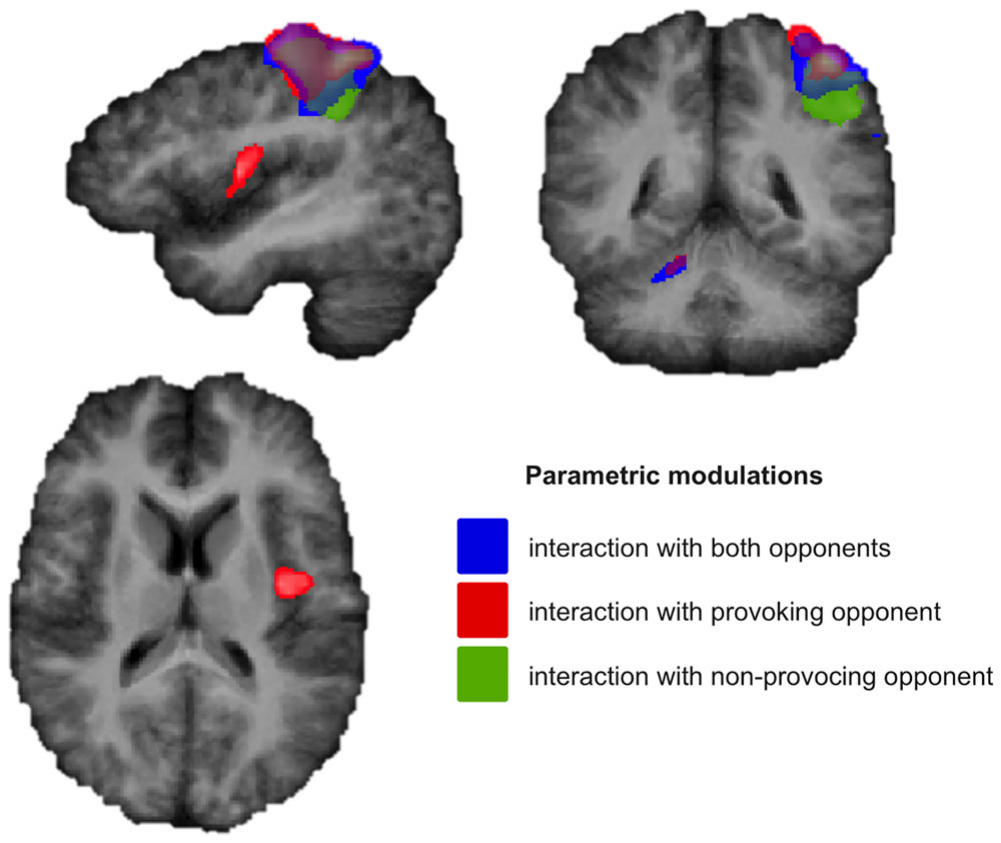
Parametric modulations. Regions modulating their activity parametrically according to the displayed behavior, when interacting with both (blue), the provoking (red), or the non-provoking opponent (green) during the Taylor Aggression Paradigm. Statistical Maps: N = 15, p < .01, Cluster Threshold level corrected, radiological convention.

Talairach coordinates of the brain regions showing parametric modulations according to the displayed behavior are reported in Table 1. Statistical maps of parametric modulations are depicted in Fig 3.

In order to identify brain regions that modulate their activity according to the displayed behavior (volume of the aversive noise chosen for the opponent defined on an 8-point scale), we conducted additional analyses in which the predictors were weighted according to this behavior. During retaliation, activity in the left pre- and post-central gyri, thus motor activation associated to the movement of the right hand was most prominent (main effect of retaliation against provoking opponent, non-provoking opponent, or both ^ parametric effect of retaliation against provoking opponent, non-provoking opponent, or both; conjunction). When interacting with the provoking opponent, the left posterior insular cortex was modulated by the intensity of retaliatory aggression displayed towards the provoking opponent (main effect of retaliation against provoking opponent ^ parametric effect of retaliation against provoking opponent; conjunction). This could not be observed for the interaction with the non-provoking opponent. Finally, activation in the right cerebellum was parametrically modulated by retaliatory aggression when interacting with both the non-provoking and the provoking opponent (main effect of retaliation against provoking opponent or both opponents ^ parametric effect of retaliation against provoking opponent or both opponents; conjunction).

During the outcome phase no significant parametric modulations due to the amount of retaliatory aggression displayed by the participants could be observed (main effect all win trials, win trials against provoking opponent, or win trials against non-provoking opponent ^ parametric effect all win trials, win trials against provoking opponent, or win trials against non-provoking opponent; conjunction).

## Discussion

The current study investigated the role of the insular cortex during retaliation and revealed that the left posterior insular cortex is specifically activated when interacting with a provoking—as opposed to a non-provoking—opponent.

### The role, segregation, and lateralization of insular activation during retaliation

During an aggressive reaction to provocation mainly bilateral insular cortex and basal ganglia were activated. This replicates results described in previous work associating insular cortex with aggression and the processing of negative emotions [9,23]. Our study provides direct quantitative support for the notion that the insular cortex is playing a crucial role in aggressive behavior: We demonstrate that insular cortex activity is parametrically modulated by the level of aggression displayed when interacting with the provoking opponent; this means that the stronger the retaliation in highly provocative situations the more insular cortex is recruited. Note that our results cannot differentiate between mere provocation and actual retaliatory aggression, as the two always occur in combination during our paradigm. This would also be the case in most real life situation involving retaliation.

We could confirm our specific hypothesis that activation within the insular cortex related to retaliation is left-lateralized and mainly localized in the posterior segment: Although the entire insular cortex was activated during retaliation (group analyses), it was specifically the activation level of the left posterior insular cortex that varied with the amount of aggression displayed. This indicates functional involvement, lateralization, and segregation of the insular cortex specific to retaliation.

Craig [12] suggested, that the insular cortex is structured along a posterior-to-anterior gradient representing the progressive integration of bodily feelings. He argued that acute emotions or interoceptive components might be represented in the posterior parts of the insular cortex, while the anterior parts seem to code for more abstract and highly integrated constructs. Retaliation or reactive aggression trigger intense bodily sensation and immediate action tendencies and, thus, should activate the posterior insular cortex. A similar segregation of insular cortex was demonstrated for a concept rather opposite to aggression, namely love. While passionate love, which is closely related to intense body sensation and action-oriented, involves posterior parts of the insular cortex, companionate love involves more anterior parts [24,25].

Although these findings are consistent with Craig’s [12] view and shed light on the neural correlates of rather abstract concepts such as aggression and love, the question remains in how far insular involvement is specific to any of these functions. In fact, the insular cortex has been associated with even more functions that are seemingly contradictory to what is reported here such as self-awareness, motor inhibition, processing of positive emotions, processing of negative emotions, and others (for review see Craig 2009). In the context of cognitive control, the insular cortex has furthermore been described as a region modulating with stimulus saliency or urgency [26–28]. However, instead of focusing on rather isolated single processes, as often done in functional brain research, social and emotional contexts should be taken into account when explaining the functional roles of brain regions. For instance, Reynolds and Berridge [29] demonstrated that varying emotional environments retunes the function of neural populations. They showed that neurons in the nucleus accumbens of rats encode alternately for fear or pleasure depending on the environment the animal is exposed to (home-like, versus low stress, versus high stress). This is a revolutionary finding, potentially suggesting that neural components alter their functional involvement according to the social situation in which they are recruited. The posterior insular cortex might not be exclusively involved in aggression, passionate love, or other concepts. Rather, it might be highly relevant in different circumstances of intense emotions which require consequent behavioral responses. Most probably, it adapts its function to whatever requirements have to be met. Further research, involving methodology reaching beyond hemodynamic neuroimaging techniques and taking into account varying emotional environments, is needed. Moreover, it should be noted that the insular cortex is highly interconnected with various brain regions and it will be of interest to investigate its specific functional interactions with those regions during different emotional and social contexts.

### Further activation during the decision phase

The insular cortex was not the only brain region activated during the decision phase; activation in superior temporal gyrus and primary motor cortex was also detected.

Activation in the right superior temporal gyrus was detected, when reacting mildly to the non-provoking opponent. This brain region has been associated with processes linked to social cognition [30–32]. Such processes are expected to be active when the participant is confronted with the non-provoking opponent; compared to his mean companion, he is friendly, nice, and from the participants’ point of view more understandable and accessible, thus an object for self-identification.

Additionally, primary motor cortex activity was detected during the decision phase. Low punishment levels (1,2,3,4) had to be selected by the left hand (leading to activation in the right primary motor cortex), whereas high levels of punishment (5,6,7,8) had to be selected by the right hand (leading to activation in left primary motor cortex). This mechanism is mirrored in our results: In the group analyses, the left motor cortex is activated during aggressive reactions towards the provoking opponent, while the right motor cortex is activated during non-aggressive reactions to the non-provoking opponent. Accordingly, activity in the right motor cortex modulated with the intensity of the chosen punishment independent of the provocation condition; the higher the chosen punishment, the more involvement of the left motor cortex was observed. It has to be emphasized that further left-lateralizations (e.g. of anterior insula activation) could also be interpreted in light of this motor dissociation.

### Winning and losing

Winning was associated with vast neural activity in bilateral superior and middle frontal regions, the inferior parietal lobes, the left precuneus, and bilateral striatum. This replicates previous results [9]. Striatal activation on one hand and the involvement of prefrontal areas on the other hand, strongly suggest the recruitment of the reward circuit in the brain [33]. Winning during the TAP is rewarding in two ways: Outperforming the opponent in the given task (reaction time competition) might be rewarding in itself. Furthermore, winning means avoiding suffering from retaliatory aggression and at the same time administering retaliatory aggression to the opponent, which might also be a pleasurable experience.

In contrast, losing was associated with bilateral activation in the superior temporal gyri and the parahippocampal gyri. The former might simply reflect the anticipated auditory stimulus, which is to come every time a participant loses (and never, when the participant wins). Previously, the parahippocampal gyri were shown to be involved in scene recognition and the detection of paralinguistic speech profiles often related to the social component of the situation (e.g. sarcasm [34]). This might reflect the paralinguistic and socially driven interpretation of the communication during the outcome phase; although the information presented during this phase is objective and seemingly neutral (“you won / you lost” and “your opponent chose x”), it contains social and paralinguistic cues related to the perception of the social opponent and the interpretation of his behavior.

The only opponent-specific activation regarding the outcome phase was detected when losing against the non-provoking opponent instead of the provoking opponent. When no highly aversive stimulus had to be expected, the parietal lobes and the left middle temporal gyrus were significantly activated. The participant might feel relief, when losing against the non-provoking opponent instead of the provoking opponent, as the noise feedback which is about to come is much less aversive. However, an association between the detected brain regions and the described processes has not been investigated yet.

Generally, the neural correlates of winning and losing were not linked to the individual levels of displayed aggression; no parametric modulations in any brain regions could be detected during the outcome phase.

## Limitations

The current results have to be interpreted considering the limitations of our experimental design: The main limitation of this study is the rather small sample size (N = 15) which makes the representativeness of our findings for the general male population challenging. We suggest continuing the investigation of social interaction by means of functional imaging based on larger samples (e.g., [11]). Furthermore, only healthy, young, male students were examined rendering the generalization of our results to a gender-unspecific context or any clinical population impossible. One should also consider that the concrete implementation of the Taylor Aggression Paradigm in our study limited the variety of observable behavior. Firstly, participants did not react aggressively towards the non-provoking opponent making any analysis of proactive aggression and comparisons between proactive (non-provoked) and retaliatory aggression impossible. Secondly, in our paradigm mere provocation and the actual retaliation behavior could not be differentiated. Our interpretations are based on an understanding of retaliation and the provocative situation as a unity, thus, as one holistic social situation. Therefore, the interpretations resulting from the presented findings definitely lack in specificity. However, as pointed out previously [8], it is unclear whether identifying neural components exclusively involved in the perception of provocation versus actual retalialiatory behavior would ultimately lead to a better understanding of real life aggression. In a naturalistic setting both concepts always co-occur. Finally, the handedness of our participants was not considered which might confound our interpretations, future studies in the field should take handedness in to account.

## Conclusion

Replicating previous results in the field, this study demonstrates the crucial role of insular cortex in retaliation. We show that the left posterior insular cortex is a core brain region involved in retaliatory aggression; this was specifically demonstrated for provocative versus non-provocative social interactions. We employed random effects group analyses and examined parametric modulations of brain activity during a controlled behavioral aggression paradigm. The left-lateralization of insular activity during retaliation is in line with evidence from electro-physiological studies, suggesting left-lateralized fronto-cortical dominance during anger processing and aggressive acts [14]. Furthermore, our results support the theory that particularly the posterior segment of insular cortex is involved in the processing of emotions triggering intense bodily sensations and immediate action tendencies [12].
